# S-Layer Protein of *Lactobacillus helveticus* SBT2171 Promotes Human β-Defensin 2 Expression via TLR2–JNK Signaling

**DOI:** 10.3389/fmicb.2019.02414

**Published:** 2019-10-18

**Authors:** Eiji Kobatake, Toshihide Kabuki

**Affiliations:** Milk Science Research Institute, Megmilk Snow Brand Co., Ltd., Saitama, Japan

**Keywords:** surface-layer protein, antimicrobial peptide, human β-defensin 2, lactic acid bacteria, *Lactobacillus helveticus* SBT2171, c-Jun N-terminal kinase, toll-like receptor 2

## Abstract

Antimicrobial peptides that contribute to innate immunity are among the most important protective measures against infection in many organisms. Several substances are known to regulate the expression of antimicrobial peptides. In this study, we investigated the factors in lactic acid bacteria (LAB) that induce antimicrobial peptide expression in the host. We found that *Lactobacillus helveticus* SBT2171 (LH2171) induced the expression of human β-defensin (hBD)2 in Caco-2 human colonic epithelial cells. Specifically, surface layer protein (SLP) of LH2171 stimulated hBD2 expression by activating c-Jun N-terminal kinase (JNK) signaling via Toll-like receptor (TLR)2 in Caco-2 cells. SLPs extracted from other lactobacilli similarly increased hBD2 expression, suggesting that this stimulatory effect is common feature of *Lactobacillus* SLPs. Interestingly, *Lactobacillus* strains that strongly induced hBD2 expression also potently activated JNK signaling. Thus, upregulation of hBD2 induced by TLR2–JNK signaling contributes to protection of the host against infection.

## Introduction

Organisms are constantly exposed to infectious agents in the environment. To protect against infection, organisms have evolved defense strategies including immune responses. The innate immune system rapidly and non-specifically eliminates foreign substances such as pathogens from the body and is therefore considered as the first line of host defense. This system comprises cellular and humoral components; the latter includes antimicrobial peptides, which are found in mammals, amphibians, and insects and exert bactericidal effects mainly by directly interacting with target bacteria and perturbing cell membrane function (Diamond et al., 2008). These peptides exhibit cytotoxic activity not only against a broad spectrum of bacteria, but also against fungi and viruses (Dale and Fredericks, 2005), and can therefore serve as effective and safe antibiotics.

Defensins are the major type of antimicrobial peptide in humans and play important roles in host defense. Specifically, β-defensins (BD) are distributed in the mucosal epithelium and skin, which are in direct contact with the external environment (Ganz, 2003). Human (h)BD1, hBD2, and hBD3 have been extensively studied; hBD2 and hBD3 are induced by inflammation or bacteria (Harder et al., 2001; Ganz, 2003), whereas hBD1 is constitutively expressed. For example, vitamin D (De Filippis et al., 2017), ceramide-1-phosphate (Kim et al., 2014), various plant secondary metabolites (Schwab et al., 2008; Lombardo Bedran et al., 2014; Xiong et al., 2015), and bacterial factors (Ogushi et al., 2001) are known to induce BD expression in epithelial cells. Thus, increasing BD expression is expected to prevent infections.

Lactic acid bacteria are popular food ingredients with known health benefits. Many strains of LAB are known as probiotics, which are defined as “live microorganisms that, when administered in adequate amounts, confer health benefits on the host” (FAO/WHO, 2002), and act primarily in the gut where they improve gut health. Additionally, some bacterial components are bioactive substances—known as paraprobiotics, which are defined as ‘non-viable microbial cells (intact or broken) or crude cell extracts (i.e., with complex chemical composition), which, when administered (orally or topically) in adequate amounts, confer a benefit on the human or animal consumer’ (Taverniti and Guglielmetti, 2011), or biogenics, which are “food ingredients which beneficially affect the host by directly immunostimulating or suppressing mutagenesis, tumorigenesis, peroxidation, hypercholesterolemia or intestinal putrefaction” (Mitsuoka, 2000).

Several strains of LAB are known to induce BD expression (Schlee et al., 2008; Zhang et al., 2011). We have also confirmed that *L. helveticus* SBT2171 (LH2171) induces the expression of BDs in epithelial cells (Kobatake et al., 2019). We speculated that this effect was exerted by bioactive components of LH2171 that act as paraprobiotics or biogenics. To test this hypothesis, in the present study, we identified the bacterial components of LH2171 that enhance BD levels and investigated their functions.

## Materials and Methods

### Bacterial Strains

*Lactobacillus helveticus* SBT2171 (LH2171) and *L. brevis* SBT10966 were isolated by Megmilk Snow Brand (Tokyo, Japan). LH2171 was deposited in the International Patent Organism Depositary, National Institute of Technology and Evaluation (Chiba, Japan) under the accession number FERM BP-5445. *L. helveticus* JCM1120^T^ (equivalent to ATCC 15009, type strain), *L. acidophilus* JCM1132^T^ (equivalent to ATCC 4356, type strain), *L. amylovorus* JCM1126^T^ (equivalent to ATCC 33620, type strain), and *L. buchneri* JCM1115^T^ (equivalent to ATCC 4005, type strain) were purchased from Japan Collection of Microorganisms (Tsukuba, Japan).

### Preparation of LAB

Lactic acid bacteria were cultivated at 37°C in De Man, Rogosa, and Sharpe (MRS) broth (Difco, Detroit, MI, United States) for 16 h and were harvested by centrifugation at 8,000 × *g* for 10 min at 4°C. The cell pellet was washed twice with phosphate-buffered saline (PBS) and once with sterile distilled water, then resuspended in distilled water and lyophilized. LAB powder was resuspended in PBS and used for assays.

### SLP Extraction and Purification

Surface layer protein was extracted from bacterial cells and purified as previously described (Konstantinov et al., 2008; Taverniti et al., 2013), with several modifications. Bacteria were cultivated at 37°C in MRS broth for 16 h and harvested by centrifugation at 8,000 × *g* for 10 min at 4°C. The harvested cells were washed once with sterile distilled water and the cell pellet was resuspended in 1 M LiCl solution containing complete Protease Inhibitor Cocktail (Roche, Roswell, GA, United States), and SLP was extracted for 30 min at room temperature with gentle agitation. The cells were collected by centrifugation at 10,000 × *g* for 10 min at 4°C and SLP was extracted with 5 M LiCl solution containing complete Protease Inhibitor Cocktail for 30 min at room temperature. The collected supernatants were combined and passed through a 0.2-μm pore size membrane filter, and dialyzed for 24 h at 4°C against distilled water using Slide-A-Lyzer G2 Dialysis Cassettes 10K (Thermo Fisher Scientific, Waltham, MA, United States). The dialysate was collected and centrifuged at 12,000 × *g* for 20 min at 4°C. The pellet was washed with sterile distilled water, resuspended in 1 M LiCl solution, and stirred for 15 min on ice. The pellet was collected by centrifugation, washed with distilled water, and then lyophilized to obtain purified SLP. The weight of purified SLP was measured and SLP suspension in PBS was conditioned to use for assay. To obtain SLP-deficient LAB, bacterial cells treated with LiCl solution were collected, washed once with sterile distilled water, and then lyophilized.

### Digestion of SLP

Surface layer protein was resuspended in PBS (5 mg/ml) and proteinase K (from *Tritirachium album*; Sigma-Aldrich, St. Louis, MO, United States) was added at a final concentration of 100 μg/ml, followed by incubation at 37°C for 16 h. The proteinase K was deactivated by heating at 96°C for 10 min.

### DNA Extraction

Lactic acid bacteria were cultivated at 37°C in MRS broth for 16 h and harvested by centrifugation at 8,000 × *g* for 10 min at 4°C. The cell pellet was washed once with PBS and DNA was extracted from the cells using the MORA-EXTRACT kit (Kyokuto Pharmaceutical Industrial Co., Tokyo, Japan) according to the manufacturer’s instructions.

### CW and PGN Preparation

CW and PGN were prepared from cells as previously reported (Hatakeyama et al., 2011), with several modifications. Lyophilized LAB powder was resuspended in PBS and disrupted four times using a French press (Ohtake, Tokyo, Japan) at a pressure of 180 MPa. After centrifugation of the mixture at 50,000 × *g* for 10 min at 4°C, the pellet was resuspended in PBS and centrifuged at 500 × *g* for 1 min at 4°C to remove undisrupted cells. The supernatant was collected and centrifuged at 10,000 × *g* for 15 min at 4°C. The pellet was washed with sterile distilled water and lyophilized. This fraction was resuspended in 4.5% SDS solution and heated at 100°C for 1 h, then washed three times with distilled water to remove all traces of SDS. The pellet was incubated in 30 μg/ml pronase (from *Streptomyces griseus*; Roche)/10 mM phosphate buffer (pH 7.0) at 37°C for 1 h. The pellet was collected by centrifugation (10,000 × *g*, 20 min, 20°C) and washed twice with distilled water. The pellet was incubated in 10 μg/ml DNase I (from bovine pancreas; Roche)/10 μg/ml RNase A (Thermo Fisher Scientific)/10 mM phosphate buffer (pH 7.0) at 37°C for 1 h. The pellet was collected by centrifugation and washed twice with distilled water, then incubated in 100 μg/ml pepsin (from porcine gastric mucosa; Sigma-Aldrich)/0.02N HCl at 37°C for 20 h. The pellet was collected by centrifugation and washed five times with distilled water, lyophilized, and used as CW. To obtain purified PGN, CW was incubated in 10% trichloroacetic acid at 80°C for 10 min, collected by centrifugation, and washed with distilled water. The pellet was incubated with 100 μg/ml pronase/10 mM phosphate buffer (pH 7.0) at 37°C for 6 h, collected by centrifugation, and washed three times with distilled water. The pellet was lyophilized and used as PGN.

### Cell Culture

Caco-2 cells were purchased from the American Type Culture Collection (Manassas, VA, United States) and cultured in high-glucose Dulbecco’s modified Eagle’s medium (DMEM) (Wako, Osaka, Japan) supplemented with 10% fetal bovine serum, 1 × minimal essential medium non-essential amino acids, 100 U/ml penicillin, and 100 μg/ml streptomycin at 37°C and 5% CO_2_. HSC-4 cells were obtained from the Japanese Collection of Research Bioresources Cell Bank (Health Science Research Resources Bank, Osaka, Japan) and cultured in DMEM supplemented with 10% fetal bovine serum, 100 U/ml penicillin, and 100 μg/ml streptomycin at 37°C and 5% CO_2_.

### *In vitro* Gene Expression Assay

Caco-2 cells were seeded in 12-well plates (2 × 10^5^ cells/well) and HSC-4 cells were seeded in 24-well plates (1 × 10^5^ cells/well). The cells were incubated overnight at 37°C and 5% CO_2_. The next day, the medium was changed to DMEM without serum and antibiotics followed by incubation for 24 h. The cells were then treated with SLP for 6 h, washed with PBS, and total RNA was extracted using the RNeasy Mini Kit (Qiagen, Hilden, Germany). First-strand cDNA synthesis was performed using the ReverTra Ace qPCR RT Master Mix with gDNA Remover (Toyobo, Osaka, Japan) according to the manufacturer’s instructions. Real time-PCR analysis was carried out using TaqMan Fast Advanced Master Mix (Applied Biosystems, Foster City, CA, United States). The following TaqMan Gene Expression Assays were used: hBD2 (Hs00175474_m1) and glyceraldehyde 3-phosphate dehydrogenase (GAPDH; Hs03929097_g1). The relative expression level of hBD2 mRNA was calculated with the ΔΔCt method and normalized to that of GAPDH (endogenous control).

### TLR2 Inhibition Assay

The inhibition assay with TLR2-neutralizing antibody was carried out as previously described (Taverniti et al., 2013), with several modifications. Caco-2 cells were seeded in 12-well plates (2 × 10^5^ cells/well) and incubated at 37°C and 5% CO_2_. The next day, the medium was changed to DMEM without serum and antibiotics followed by incubation for 24 h. The cells were then treated with anti-TLR2 antibody (Invivogen, San Diego, CA, United States) for 1 h. In accordance with the manufacturer’s instructions, a human immunoglobulin A2 (IgA2) isotype (Invivogen) was used as a control to prevent non-specific binding and blocking activities of the antibody. Each antibody was used at 5 μg/ml. After treatment, SLP (final concentration: 10 μg/ml) was added to the cells, followed by incubation for 6 h. Gene expression was measured as described above.

### MAPK and NF-κB Inhibition Assay

Caco-2 cells were seeded in 12-well plates (2 × 10^5^ cells/well) and incubated at 37°C in 5% CO_2_. The next day, the medium was changed to DMEM without serum and antibiotics, followed by incubation for 24 h. The cells were then treated with 20 μM inhibitor (SB202190 for p38, SP600125 for JNK, PD98059 for ERK, and JSH-23 for NF-κB) for 1 h. After treatment, SLP (final concentration: 10 μg/ml) was added to the cells, followed by incubation for 6 h. Gene expression was measured as described above.

### SDS–PAGE

Cell lysates were mixed with 2 × sample buffer and heated at 95°C for 5 min, and proteins were separated on a 4–20% Mini-PROTEAN TGX Precast Gel (Bio-Rad, Hercules, CA, United States) that was then stained with Quick-CBB (Wako).

### Western Blotting

Caco-2 cells were seeded in 6-well plates (4 × 10^5^ cells/well) and incubated at 37°C and 5% CO_2_. The next day, the medium was changed to DMEM without serum and antibiotics, followed by incubation for 24 h. The cells were treated with SLP (10 μg/ml) for 1 h. For the TLR2 inhibition assay, cells were pretreated with anti-TLR2 antibody or human IgA2 isotype (5 μg/ml) for 1 h before SLP treatment. The cells were then washed with PBS and lysed in radioimmunoprecipitation assay buffer (50 mM Tris [pH 7.5], 150 mM NaCl, 1% Non-idet P-40, 0.5% deoxycholic acid, and 0.1% SDS) supplemented with complete protease inhibitor cocktail and PhosSTOP phosphatase inhibitor cocktail (Roche), followed by incubation at 4°C for 1 h. The residue in cell lysates was removed by centrifugation at 7,500 × *g* for 5 min at 4°C. Proteins were separated on a 12% Mini-PROTEAN TGX Precast Gel (Bio-Rad) and transferred to an Immobilon-P membrane (Millipore, Billerica, MA, United States) that was blocked with Western BLoT Blocking Buffer (Protein-free) (Takara Bio, Otsu, Japan) or 5% bovine serum albumin in Tris-buffered saline containing 0.1% Tween 20 (TBS-T) for 1 h at room temperature, then incubated overnight at 4°C with primary antibodies against the following proteins: phospho-JNK Thr183/Tyr185 (81E11), JNK, phospho-c-Jun Ser73 (D47G9), c-Jun (60A8), and β-actin (13E5) (all from Cell Signaling Technology, Danvers, MA, United States). The membrane was washed with TBS-T and incubated for 1 h at room temperature with horseradish peroxidase (HRP)-conjugated anti-rabbit IgG (Cell Signaling Technology) as the secondary antibody. HRP signals were visualized using the Immobilon Western chemiluminescent HRP substrate (Millipore) and ChemiDoc MP Imaging System (Bio-Rad). Band intensity was quantified with Image Lab software (Bio-Rad).

### N-Terminal Sequence Analysis of SLP

N-terminal sequence analysis of SLP was performed by Nippi (Tokyo, Japan).

### Expression and Purification of Recombinant SLP

Recombinant SLP was expressed in *Escherichia coli* and purified by SignalChem (Richmond, BC, Canada). The mature SLP gene was amplified by PCR from LH2171 DNA with the following primers: 5′-ggtggatccgctatgccagttaacgctgctact-3′ (forward) and 5′-ctcgaattcagtcaaagttttcaactctaacgtaagtc-3′ (reverse). The amplified fragments were cloned into pET22b plasmid (SignalChem), which added an N-terminal His tag to the protein. The plasmid was transformed into *E. coli* BL21 (DE3) by the standard heat shock technique and protein expression was induced with isopropyl-β-D-thiogalactopyranoside. His-tagged SLP was purified and the protein concentration was determined from Coomassie blue-stained SDS–PAGE gels.

### Phylogenetic Analysis

Multiple sequence alignment of primary amino acid sequences was performed using Clustal Omega (Larkin et al., 2007), and the phylogenetic tree was drawn using TreeView X software (Softonic, Barcelona, Spain).

### Statistical Analysis

Data are expressed as mean ± standard deviation. Differences between groups were evaluated by one-way analysis of variance (ANOVA) and the Tukey–Kramer *post hoc* test or by one-way ANOVA and Dunnett’s *post hoc* test for multiple comparisons, and with the Student’s t test for single comparisons. A *P*-value < 0.05 was considered statistically significant.

### Accession Numbers

The reported sequence data of LH2171 *slpH* gene are available in the DNA Data Bank of Japan database under accession number LC441113^1^.

## Results

### Surface Layer Protein Contributes to the Enhancement of hBD2 Expression by LH2171

**FIGURE 1:**
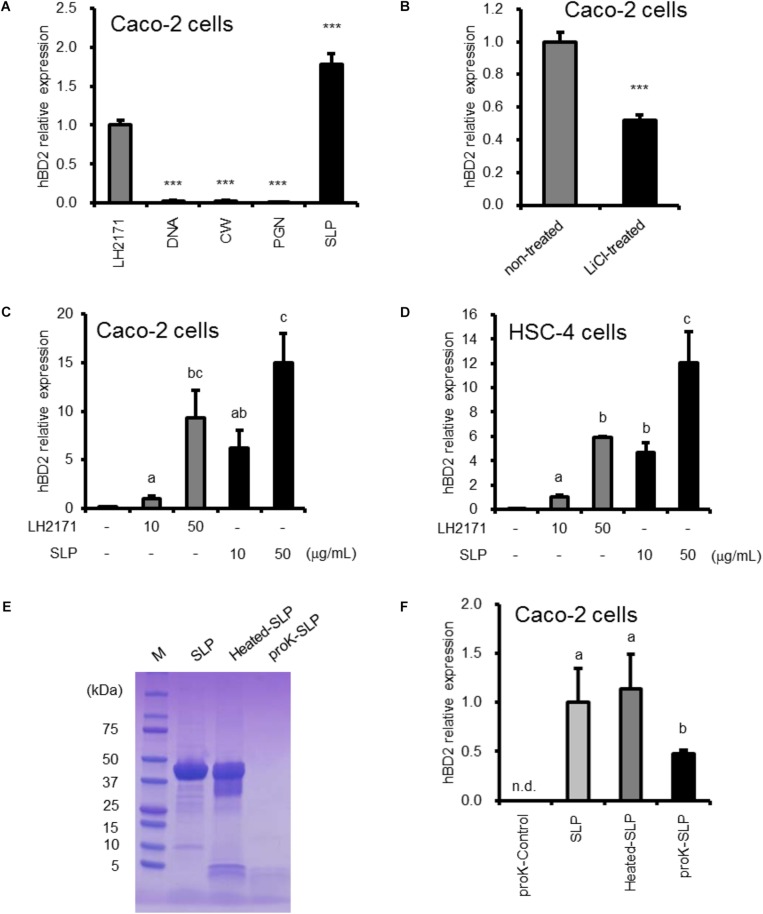
hBD2 upregulation in epithelial cells by strain LH2171 and its components. Cells were cultured with LH2171 or its cellular components [DNA, cell wall (CW), peptidoglycan (PGN), Surface layer protein (SLP)] **(A,C,D)**, with LH2171 (non-treated) or SLP-deficient LH2171 (LiCl-treated) **(B)**, and with SLP, heated SLP, or proteinase K-treated SLP (proK-SLP) **(F)**. hBD2 mRNA levels were evaluated by quantitative real time-PCR. Each experiment was performed in triplicate; data are shown as mean ± SD **(A–D,F)**. Samples were added at a concentration of 10 μg/ml **(A,B,F)**. ^∗∗∗^*P* < 0.001 vs. LH2171 (one-way ANOVA and Dunnett’s *post hoc* test) **(A)**. ^∗∗∗^*P* < 0.001 according to the Student’s *t*-test **(B)**. Values not sharing a common letter are significantly different (*P* < 0.05 by one-way ANOVA and Tukey–Kramer *post hoc* test) **(C,D,F)**. SDS–PAGE profile of digested SLP **(E)**.

To identify the bacterial factors that induce BD expression, we purified several bacterial components from LH2171 and evaluated their effects on Caco-2 human colonic epithelial cells *in vitro*. We found that SLP increased hBD2 expression to a greater extent than LH2171 bacterial cell (Figure 1A). In this study, we investigated hBD2 expression in undifferentiated Caco-2 cells, hence the results of hBD2 expression assays in Caco-2 cells did not changed basically depending on the differentiation state (Supplementary Figure S1). Furthermore, LH2171 depleted of SLP by lithium chloride treatment showed a weaker inductive effect than untreated LH2171 (Figure 1B). The effect of lithium chloride on hBD2 expression did not observed (Supplementary Figure S2). The upregulation of hBD2 mRNA by SLP was dose dependent (Figure 1C). LH2171 SLP had a similar effect on hBD2 expression in HSC-4 human tongue epithelial cells (Figure 1D). In contrast, other cellular components such as DNA, CW, and PGN purified from LH2171 had no effect on hBD2 level.

We also investigated whether SLP digestion results in the loss of activity. Proteinase K digested nearly all of LH2171 SLP, which did not occur by heating at 96°C for 10 min (Figure 1E). The *in vitro* assay revealed that proteinase K digestion decreased hBD2 expression induced by SLP (Figure 1F), suggesting that SLP mediates the stimulatory effect of LH2171 on hBD2 expression.

### Characterization of SLP Purified From LH2171

**FIGURE 2:**
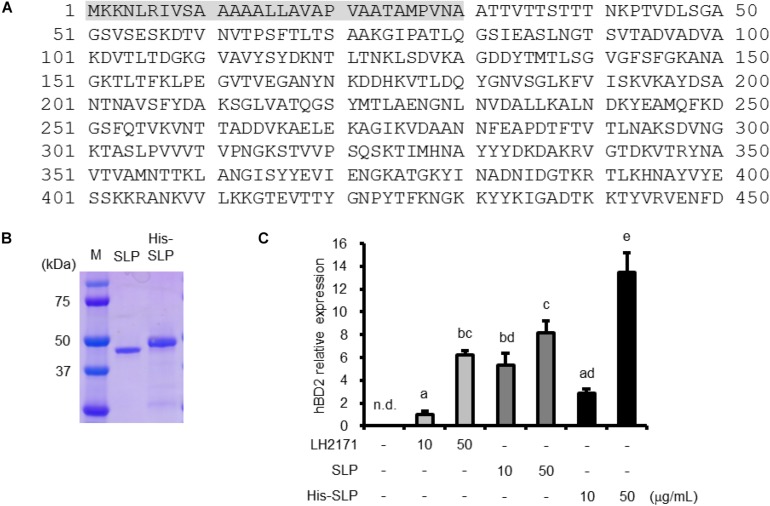
Characterization of LH2171 SLP. Deduced amino acid sequence of LH2171 SLP (full length). Shaded area (residues 1–30) represent the signal peptide **(A)**. SDS–PAGE profile of purified and recombinant SLP **(B)**. Caco-2 cells were cultured with LH2171 or SLP. hBD2 mRNA levels were evaluated by quantitative real time-PCR. Each experiment was performed in triplicate; data are shown as mean ± SD. Values not sharing a common letter are significantly different (*P* < 0.05 by one-way ANOVA and Tukey–Kramer *post hoc* test) **(C)**.

We analyzed the N-terminal sequence of SLP purified from LH2171 to characterize the molecular basis for its inductive activity. The first 16 N-terminal amino acids were ATTVTTSTTTNKPTVD (Figure 2A); an examination of the LH2171 genome confirmed that this sequence is present in the *slpH* gene of LH2171 (accession number LC441113). The full-length LH2171 SLP consists of 450 amino acids and the purified mature protein was found to lack a signal peptide (residues 1–30) (Figure 2A). We expressed recombinant SLP in *E. coli* and evaluated the effect of the purified protein on hBD2 expression in Caco-2 cells (Figure 2B). Recombinant SLP increased hBD2 mRNA level in a dose-dependent manner similar to SLP purified from LH2171 cells (Figure 2C). These results confirm that SLP is the active component of LH2171 that stimulates hBD2 expression.

### Toll-Like Receptor 2 and JNK Activation by SLP Induces hBD2 Upregulation

**FIGURE 3:**
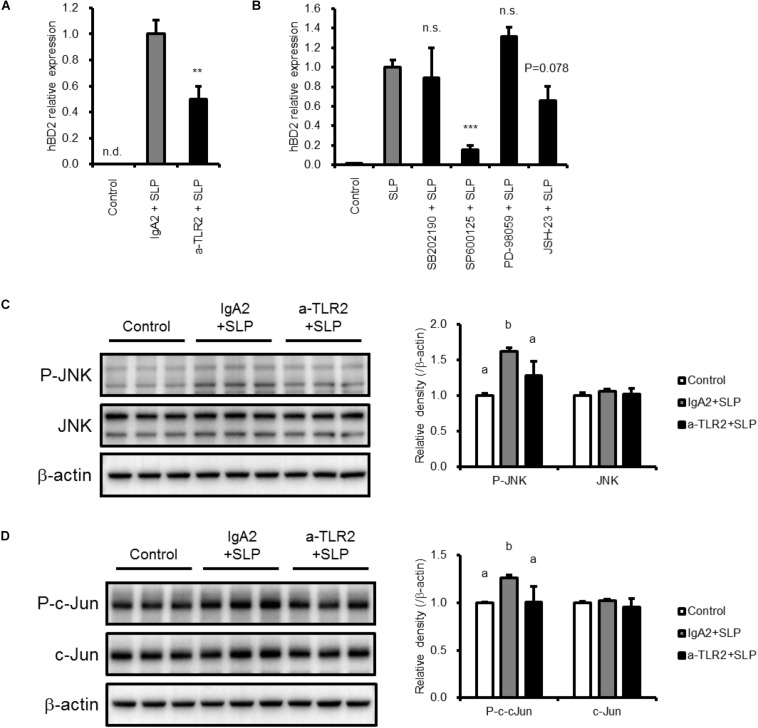
Mechanisms of hBD2 upregulation by LH2171 SLP. Caco-2 cells were pre-incubated with anti-TLR2 antibody (a-TLR2), isotype control antibody (IgA2) **(A,C,D)**, or the indicated inhibitor (SB202190 for p38, SP600125 for JNK, PD98059 for ERK, and JSH-23 for NF-κB) **(B)** and then treated with LH2171 SLP. hBD2 mRNA levels were evaluated by quantitative real time-PCR **(A,B)**, and protein levels of total JNK and phospho-JNK (P-JNK) **(C)** or total c-Jun and phospho-c-Jun **(D)** in total cell lysates were analyzed by western blotting. Each experiment was performed in triplicate; data are shown as mean ± SD **(A–D)**. ^∗∗^*P* < 0.01 and ^∗∗∗^*P* < 0.001 vs. IgA2 + SLP **(A)** or SLP **(B)** (one-way ANOVA and Dunnett’s *post hoc* test). Values not sharing a common letter are significantly different (*P* < 0.05 by one-way ANOVA and Tukey–Kramer *post hoc* test) **(C,D)**.

We next investigated the mechanism underlying the upregulation of hBD2 by LH2171 SLP. To determine whether TLR2 is involved, Caco-2 cells were pretreated with TLR2-neutralizing or isotype control antibody and incubated with LH2171 SLP. The increase in hBD2 expression induced by SLP was abrogated by TLR2-blocking antibody treatment (Figure 3A), indicating that TLR2 mediates the upregulation of hBD2 by LH2171 SLP.

To further investigate the involvement of intracellular signaling pathways in the effects of SLP, we evaluated hBD2 expression following pretreatment with inhibitors of MAPK signaling components (including p38 MAPK, JNK, and ERK) and NF-κB. Caco-2 cells pretreated with each inhibitor were incubated with LH2171 SLP for 6 h before hBD2 expression was evaluated (Figure 3B). We found that JNK inhibition by SP600125 abolished the increase in hBD2 induced by LH2171 SLP, whereas the p38 MAPK inhibitor SB202190 and ERK inhibitor PD-98059 did not have this effect. The NF-κB inhibitor JSH-23 also tended to suppress the upregulation of hBD2 but to a lesser degree than JNK inhibition. Thus, LH2171 SLP induces hBD2 expression mainly via JNK signaling.

To confirm the activation of JNK signaling in response to LH2171 SLP treatment, we examined the phosphorylation JNK and c-Jun in Caco-2 cells and found that both were increased by SLP treatment (Figures 3C,D), confirming that LH2171 SLP activates JNK signaling. Furthermore, pretreatment with TLR2-neutralizing antibody abolished JNK and c-Jun phosphorylation. Thus, LH2171 SLP stimulates TLR2 and activates downstream JNK signaling to enhance hBD2 expression.

### hBD2 Expression Is Induced by SLP of Other *Lactobacillus* Species

**FIGURE 4:**
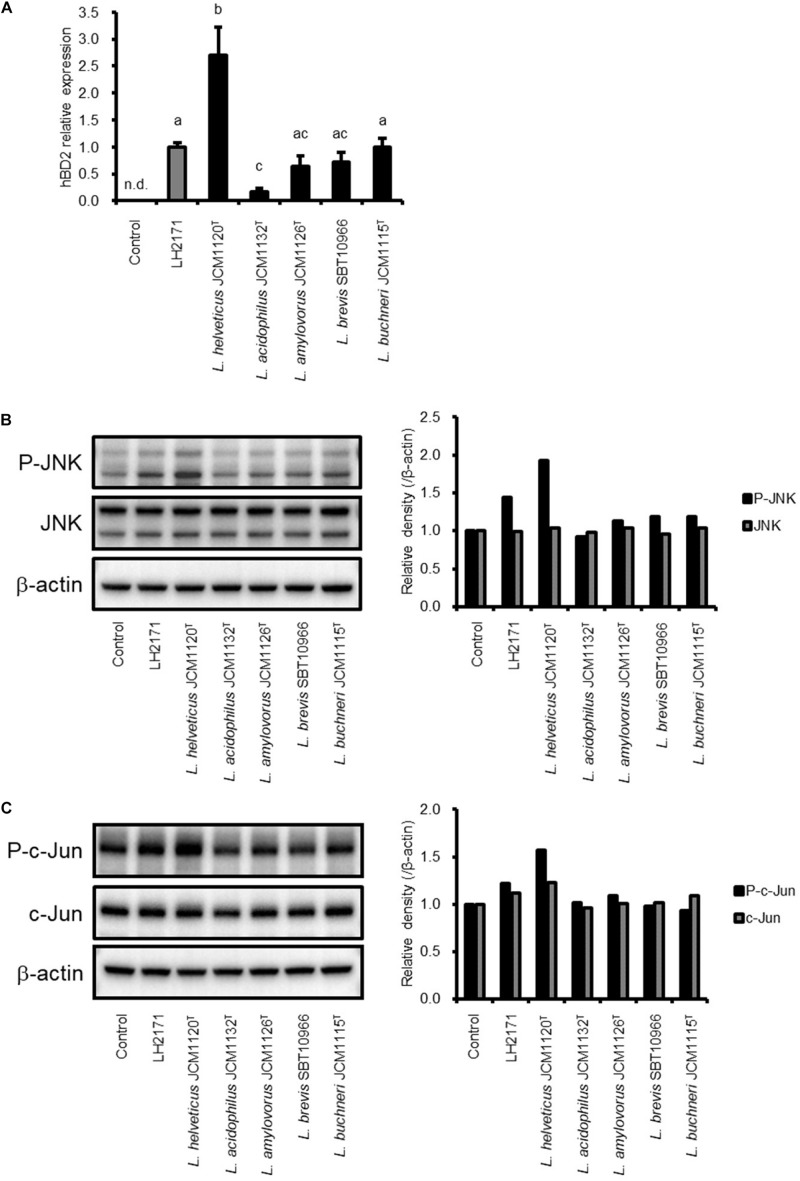
Comparison of SLP activity between LH2171 and other *Lactobacillus* strains. Caco-2 cells were cultured with each SLP, and hBD2 mRNA levels were evaluated by quantitative real time-PCR. Each experiment was performed in triplicate; data are shown as mean ± SD. Values not sharing a common letter are significantly different (*P* < 0.05 by one-way ANOVA and Tukey–Kramer *post hoc* test) **(A)**. Protein levels of total JNK and phospho-JNK (P-JNK) **(B)** or total c-Jun and phospho-c-Jun **(C)** in total cell lysates were analyzed by western blotting.

We investigated whether SLP purified from other *Lactobacillus* species have the same effect on hBD2 expression as LH2171 SLP. To this end, SLP was purified from *L. helveticus* JCM1120^T^, *L. acidophilus* JCM1132^T^, *L. amylovorus* JCM1126^T^, *L. buchneri* JCM1115^T^, and *L. brevis* SBT10966 cultures and used to treat Caco-2 cells. All of the SLPs induced hBD2 expression, which was negligible in untreated cells (Figure 4A). Even though all the *Lactobacillus* SLP tested induced hBD2 expression, the extent of induction among them was significantly different. These results indicate that upregulation of hBD2 is a common property of SLP derived from *Lactobacillus* species.

We also evaluated whether the SLP purified from these strains activates JNK signaling. Interestingly, the degree of JNK activation varied according to the protein; for instance, SLP purified from *L. helveticus* JCM1120^T^—which strongly induced hBD2 expression—potently activated JNK signaling. In contrast, JNK signaling was scarcely detected in cells treated with SLP purified from *L. acidophilus* JCM1132^T^, which only slightly increased hBD2 expression (Figures 4B,C). These data demonstrate that hBD2 upregulation by *Lactobacillus* SLP is dependent on JNK signaling.

### Phylogenetic Analysis of *L. helveticus* SLP

**FIGURE 5:**
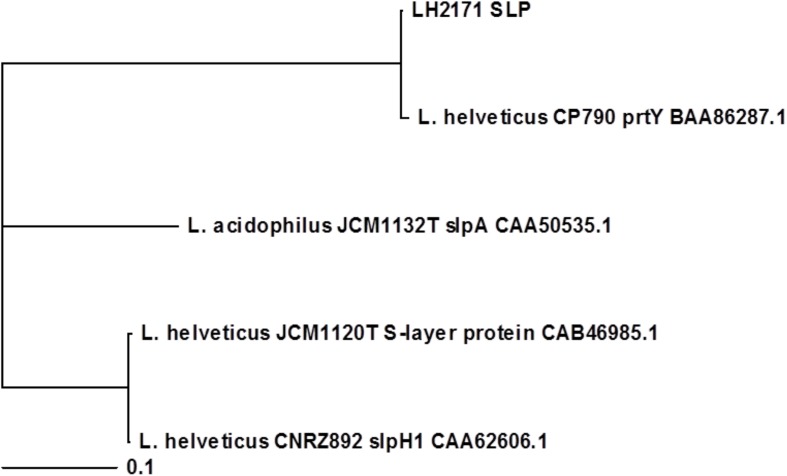
Phylogenetic analysis of *Lactobacillus* SLPs based on primary amino acid sequences. Amino acid sequences of the predicted mature forms of LH2171 SLP, *L. helveticus* JCM1120^T^ S-layer protein (accession no. CAB46985.1), *L. acidophilus* JCM1132^T^ slpA (accession no. CAA50535.1), *L. helveticus* CNRZ892 slpH1 (accession no. CAA62606.1), and *L. helveticus* CP790 prtY (accession no. BAA86287.1) were analyzed with Clustal Omega and a phylogenetic tree was constructed with TreeView X.

To investigate the relationship between the upregulation of hBD2 and SLP structure, we carried out a phylogenetic analysis of the primary amino acid sequences of SLPs from the various *Lactobacillus* species, with the sequences of *L. acidophilus* JCM1132^T^ slpA, *L. helveticus* CNRZ892 slpH1, and *L. helveticus* CP790 prtY used for comparison (Supplementary Figure S3). The results showed that LH2171 SLP and *L. helveticus* JCM1120^T^ SLP were not part of the same cluster (Figure 5), suggesting that structural differences between SLPs might be able to account for the variation in hBD2 upregulation.

## Discussion

S-layers found in many bacteria and archaea are the outermost component of CWs and are composed of SLPs that differ in terms of molecular weight and the presence or absence of post-translational modifications. SLPs bind to the CW via non-covalent bonds and cover the CW as a monomolecular layer that can be easily removed by treatment with chaotropic reagents such as LiCl, urea, and guanidinium chloride for SLP purification (Sleytr and Sára, 1997).

Several *Lactobacillus* species have been reported to express SLPs with a highly basic *pI* (higher than 9.0), which is distinct from other bacterial SLPs (Avall-Jääskeläinen and Palva, 2005; Hynönen and Palva, 2013). On the other hand, *Lactobacillus* SLPs exhibit structural diversity not only among species but also among strains. The SLPs of *L. acidophilus* and *L. helveticus* show a high degree of similarity but differ from that of *L. brevis* (Avall-Jääskeläinen and Palva, 2005). Additionally, the amino acid sequences of SLPs vary across *L. brevis* strains (Avall-Jääskeläinen and Palva, 2005; Avall-Jääskeläinen et al., 2008). SLPs of *L. kefir* and *L. parakefir* are glycosylated, while those of other *Lactobacillus* are not generally glycosylated (Mobili et al., 2009; Hynönen and Palva, 2013). These differences among *Lactobacillus* SLPs make it difficult to determine their precise functions.

In general, SLPs are thought to contribute to interactions with the external environment. *Lactobacillus* SLPs have been reported to enhance adhesion to the intestinal wall (Hynönen and Palva, 2013), protect against infection (Martínez et al., 2012; Prado Acosta et al., 2016), regulate the immune system (Konstantinov et al., 2008; Beganović et al., 2011; Taverniti et al., 2013), and exhibit an anti-oxidative function (Zhao et al., 2017). Thus, *Lactobacillus* SLPs have beneficial effects on their host as biogenics.

In this study, we showed that *Lactobacillus* SLPs stimulate the expression of antimicrobial peptides; specifically, SLP purified from *L. helveticus* strain LH2171 along with recombinant LH2171 SLP increased hBD2 expression in Caco-2 cells as well as in HSC-4 cells, another epithelial cell type. This is the first demonstration of the upregulation of an antimicrobial peptide induced by *Lactobacillus* SLPs.

It was previously reported that hBD2 expression in intestinal epithelial cells is regulated by TLR signaling (Vora et al., 2004). Furthermore, SLP of *L. helveticus* MIMLh5 contributed to target gene expression via TLR2 (Taverniti et al., 2013). Based on these observations, we speculated that LH2171 SLP is recognized by TLR2, which activates an intracellular signaling cascade that results in increased hBD2 expression; this was confirmed by our data. *L. helveticus* MIMLh5 SLP regulates the innate immune system—including the induction of tumor necrosis factor α—by stimulating TLR2 (Taverniti et al., 2013). Our data demonstrate the possibility that *Lactobacillus* SLP induce the expression of hBD2 via TLR2 and potentially regulate host innate immune response.

Lactic acid bacteria are Gram-positive bacteria that harbor lipoteichoic acids, which are the typical TLR2 agonists (Weidenmaier and Peschel, 2008). Previous report have shown that LiCl treatment does not remove lipoteichoic acids from *L. helveticus* cells (Mozes and Lortal, 1995); it is therefore presumed that SLP conditioned by LiCl treatment does not contain other TLR2 ligands. This data supports our hypothesis that LH2171 SLP promotes hBD2 expression via the TLR2 cascade.

**FIGURE 6:**
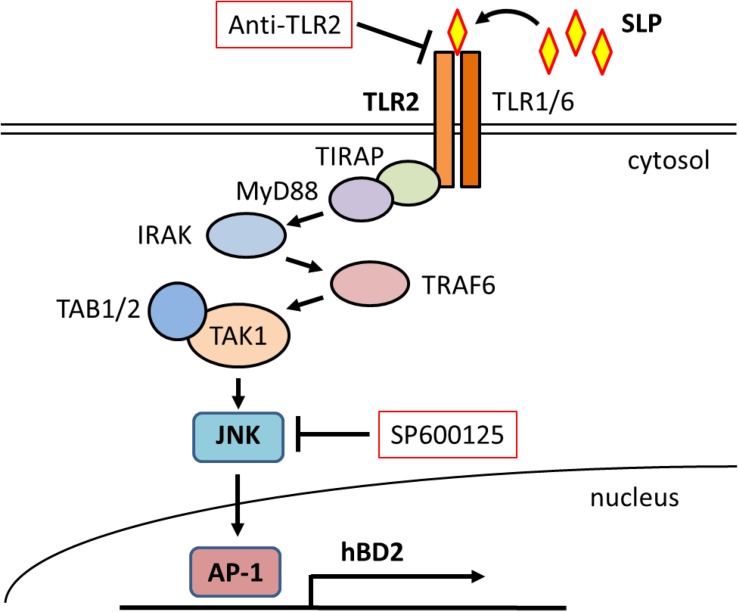
Schematic illustration of the mechanism underlying hBD2 upregulation by LH2171SLP. LH2171 SLP activates JNK signaling via TLR2 stimulation, leading to increased expression of hBD2.

TLR2 signaling induces the activation of multiple downstream pathways such as MAPK and NF-κB (Karikó et al., 2004), both of which are known to increase hBD2 expression (Ogushi et al., 2001; Vora et al., 2004; Wehkamp et al., 2004; Li et al., 2008; Xiong et al., 2015). Our results show that hBD2 level was suppressed by pretreatment with the JNK inhibitor SP600125 in LH2171 SLP-treated cells; in addition, treatment with LH2171 SLP activated JNK signaling in Caco-2 cells whereas TLR2 blockade reduced JNK signaling. Thus, LH2171 SLP activates JNK signaling via TLR2, which leads to the upregulation of hBD2 expression (Figure 6).

Cellular components of LAB are recognized by pattern recognition receptors such as TLR expressed by host cells, which activate intracellular signaling pathways (Shida et al., 2009; Kawashima et al., 2013; Kim et al., 2015; Kobatake et al., 2017). Many studies have investigated the regulation of hBD2 expression with variable findings (Ogushi et al., 2001; Vora et al., 2004; Wehkamp et al., 2004; Li et al., 2008; Xiong et al., 2015), underscoring the difficulty in establishing the beneficial effects and mechanisms of action of LAB. Our observation that *Lactobacillus* SLP induces a host defense response could contribute to the elucidation of LAB functions.

SLPs derived from five *Lactobacillus* strains (*L. helveticus* JCM1120^T^, *L. acidophilus* JCM1132^T^, *L. amylovorus* JCM1126^T^, *L. buchneri* JCM1115^T^, *L. brevis* SBT10966) increased the expression of hBD2 in Caco-2 cells, which was dependent on the degree of JNK activation. These results imply that the stimulatory effect on hBD2 is a common feature of *Lactobacillus* SLP. We speculated that structural differences among SLPs are responsible for the observed variations in hBD2 upregulation and JNK activation. *L. helveticus* JCM1120^T^ SLP more potently enhanced hBD2 expression than LH2171 SLP. *L. helveticus* SLPs form two clusters (Gatti et al., 2005; Waśko et al., 2014) with a high degree of similarity to the SLP of *L. helveticus* CNRZ892 or *L. helveticus* CP790 PrtY (Gatti et al., 2005). An amino acid sequence comparison between these SLPs and PrtY revealed that LH2171 SLP is highly similar to *L. helveticus* CP790 prtY, whereas *L. helveticus* JCM1120^T^ SLP is closely related to the *L. helveticus* CNRZ892 homolog (Table 1 and Supplementary Figure S3) (Boot et al., 1993; Callegari et al., 1998; Ventura et al., 2000; Yamamoto et al., 2000). However, *L. helveticus* JCM1120^T^ and LH2171 SLPs belong to distinct clusters (Figure 5). Thus, SLPs belonging to the group that includes *L. helveticus* JCM1120^T^ SLP may be strong inducers of hBD2 expression.

**TABLE 1:** Characteristics of analyzed proteins.

**Species**	**Strain**	**Name**	**Number of amino acid residues**		**Signal peptide**	**pI^a^**	**Similarity (%)**		**References**
							
			**Full length**	**Mature form**			**LH2171**	**JCM1120^T^**	
*L. helveticus*	SBT2171	S-layer protein	450	420	1–30	9.05	–	54.3	–
*L. helveticus*	JCM1120^T^	S-layer protein	439	409	1–30	9.21	54.3	–	Ventura et al., 2000
*L. acidophilus*	JCM1132^T^	slpA	444	420	1–24	9.49	50.0	73.5	Boot et al., 1993
*L. helveticus*	CNRZ892	slpH1	439	409	1–30	9.21	54.3	99.8	Callegari et al., 1998
*L. helveticus*	CP790	prtY	449	419	1–30	9.17	99.3	53.7	Yamamoto et al., 2000

*^*a*^Theoretical *pI* values were calculated for predicted mature proteins with the ExPASy ProtParam tool.*

Interestingly, the elevated levels of hBD2 induced by LH2171 SLP were suppressed by proteinase K digestion, but did not disappear completely (Figure 1F). A sodium dodecyl sulfate–polyacrylamide gel electrophoresis (SDS–PAGE) analysis showed that intact SLP was almost completely degraded by proteinase K treatment (Figure 1E), implying that a fragment of SLP may be sufficient to function as a TLR2 agonist and stimulate the upregulation of hBD2. In fact, protein fragments have been shown to act as TLR2 ligand and agonist; for instance, *Tannerella forsythia* BspA stimulates TLR2 and contains the sequence motif GC(S/T)GLXSIT in the leucine-rich repeat domain that is essential for this interaction (Myneni et al., 2012), while oligopeptides derived from the TLR2-activating region of *Yersinia enterocolitica* O:8 LcrV (N-terminal residues 31–57) stimulates TLR2 (Sing et al., 2002, 2005). However, the sequences reported in these previous studies are not present in LH2171 SLP, suggesting that it harbors an alternative motif that interacts with TLR2. Additionally, *Neisseria* PorB (Massari et al., 2006; Liu et al., 2010; Kattner et al., 2014), *Leptospira* LipL32 (Hsu et al., 2017), and type IIb *E. coli* enterotoxin pentameric B subunit (LT-IIb-B5) (Liang et al., 2009) have been reported as TLR2-binding proteins. Thus, TLR2 recognizes a variety of amino acid sequences. Further studies are required to determine the TLR2 recognition sites of *Lactobacillus* SLPs.

## Conclusion

In conclusion, our results reveal that *Lactobacillus* SLP increased hBD2 expression in epithelial cells and that this effect is dependent on the TLR2–JNK signaling axis. Furthermore, this stimulatory effect on hBD2 was found to be a common feature of *Lactobacillus* SLPs. Elucidating the core substructure of SLP that is recognized by TLR2 will be a focus of future work. Our findings provide evidence for a novel function of *Lactobacillus* SLP and could expand the beneficial applications of LAB.

## Data Availability Statement

The datasets generated for this study can be found in the reported sequence data of LH2171 *slpH* gene are available in the DNA Data Bank of Japan database under accession number LC441113 (http://getentry.ddbj.nig.ac.jp/getentry/na/LC441113).

## Author Contributions

EK performed the experiments. EK analyzed the data and wrote the manuscript. TK supervised the study and reviewed the manuscript. All authors contributed to the study design, and read and approved the final version of the manuscript.

## Conflict of Interest

EK and TK are employed by Megmilk Snow Brand Co., Ltd. The content of this paper was neither influenced nor constrained by this fact.

## Footnotes

1 http://getentry.ddbj.nig.ac.jp/getentry/na/LC441113

## Abbreviations

CW: cell wall
ERK: extracellular signal-regulated kinase
JNK: c-JunN-terminal kinase
LAB: lactic acid bacteria
LH2171: *Lactobacillus helveticus* SBT2171
MAPK: mitogen-activated protein kinase
NF- κ B: nuclear factor-kappa B
PGN: peptidoglycan
SLP: surface layer protein
TLR: toll-like receptor.
