# Supplementary material for: S-Layer Protein of Lactobacillus helveticus SBT2171 Promotes Human β-Defensin 2 Expression via TLR2–JNK Signaling
Source: Front Microbiol. 2019 Oct 18;10:2414. doi: 10.3389/fmicb.2019.02414 (PMC6813279; doi:10.3389/fmicb.2019.02414)
Supplement: Supplementary file 1 [file Data_Sheet_1.pdf]

Supplementary Material

S-layer protein of *Lactobacillus helveticus* SBT2171 promotes human β-defensin 2 expression via TLR2–JNK signaling

Eiji Kobatake*, Toshihide Kabuki

*** Correspondence:** Eiji Kobatake: eiji-kobatake@meg-snow.com

Supplementary Figures

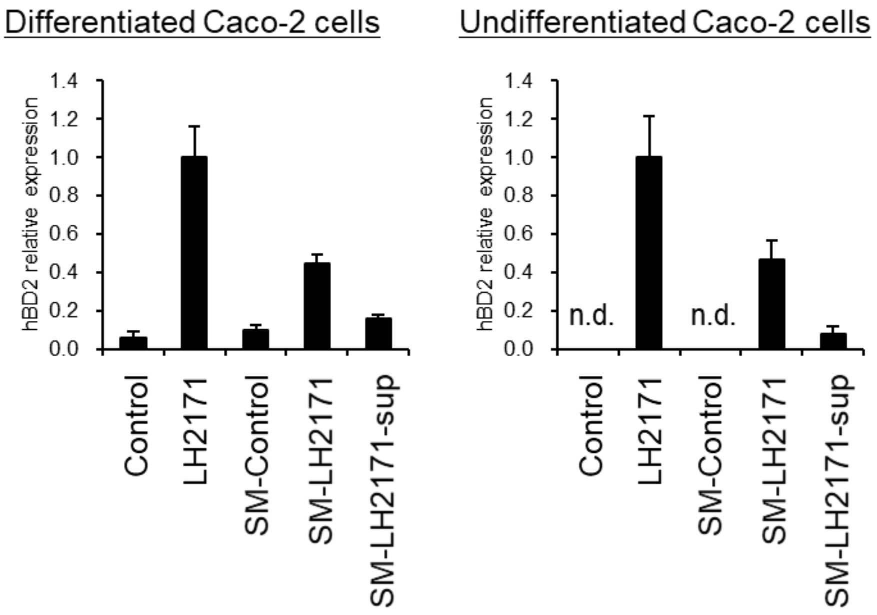

Supplementary Figure 1. The results of hBD2 expression assays in Caco-2 cells do not changed basically depending on the differentiation state. Differentiated or undifferentiated Caco-2 cells were cultured with samples shown in the following. hBD2 mRNA levels were evaluated by quantitative real time-PCR. Each experiment was performed in triplicate; data are shown as mean ± SD. Differentiated Caco-2 cells were prepared by the culturing for 21 days. (Control: medium only, LH2171: LH2171 bacterial body, SM-Control: skim milk, SM-LH2171: skim milk fermented by LH2171, SM-LH2171-sup: supernatant of skim milk fermented by LH2171)

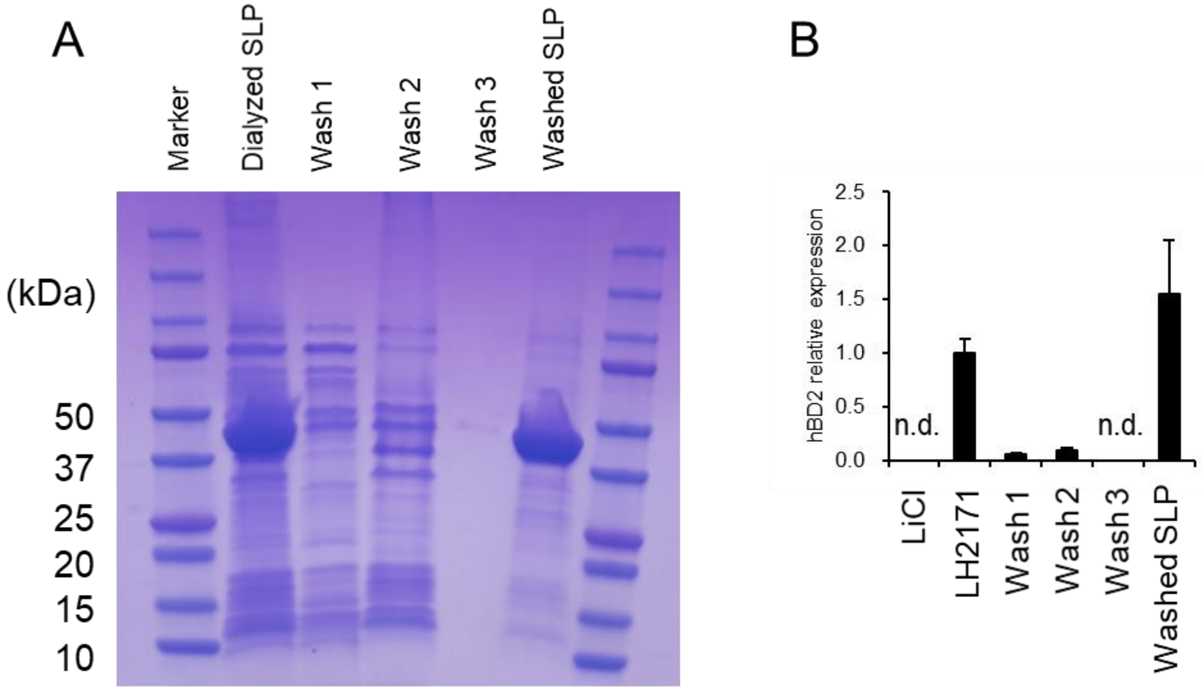

Supplementary Figure 2. Lithium chloride (LiCl) solution does not effect on hBD2 expression. The supernatants in SLP purification step were collected and evaluated in hBD2 expression assay. The dialyzed SLP was washed with distilled water (wash 1), 1M LiCl (wash 2), and distilled water (wash 3). SDS–PAGE profile of collected supernatants and washed SLP **(A)**. Caco-2 cells were cultured with LH2171, collected supernatants, or washed SLP. hBD2 mRNA levels were evaluated by quantitative real time-PCR. Each experiment was performed in triplicate; data are shown as mean ± SD. LiCl solution was used as Control **(B)**.

```
CLUSTAL O(1.2.4) multiple sequence alignment


LH2171-SLP          -ATTVTTSTTTNKPTVDLSGAGSVSESKDTVNVTPSFTL------TSAAKGIPATLQGSI 53
CP790-prtY          -ATTVTTSTTTNKPTVDLSGAGSVSESKDTVNVTPSFTL------TSAAKGIPATLQGSI 53
JCM1132T-slpA       AVSTVSAATTINASS---SAINTNTNAKYDVDVTPSVSAVAANT-ANNTPAIAGNLTGTI 56
JCM1120T-SLP        -------ATTI-NAD---SAINANTNAKYDVDVTPSISAIAAVAKSDTMPAIPGSLTGSI 49
CNRZ892-slpH1       -------ATTI-NAD---SAINANTNAKYDVDVTPSISAIAAVAKSDTMPAIPGSLTGSI 49
                           :**        *. .: :::*  *:****.:       :.   .* ..* *:*

LH2171-SLP          EASLNGTSVTADVADVAKDVTLTDGKGVAVYSYDKNTLTNKLSDVKAGDDYTMTLSGVGF 113
CP790-prtY          EASLNGTSVTADVADVAKDVTLTDGKGVAVYSYDKNTLTNKLSDVKAGDDYTMTLSGVGF 113
JCM1132T-slpA       SASYNGKTYTANLKADTENATITAAG---------STTAVKPAELAAGVAYTVTVNDVSF 107
JCM1120T-SLP        SASYNGKSYTANLPKDSGNATITDSN----------NNTVKPAELEADKAYTVTIPDVSF 99
CNRZ892-slpH1       SASYNGKSYTANLPKDSGNATITDSN----------NNTVKPAELEADKAYTVTVPDVSF 99
                    .** **.: **::   : :.*:* .           . : * ::: *.  **:*: .*.*

LH2171-SLP          SFGKANAGKTLTFKLPEGV-TVEG------ANYNKDDHKVTLDQYGNVSGLKFVISKVKA 166
CP790-prtY          SFGKANAGKTLTFKLPEGV-TVEG------ANYNKDDHKVTLDQYGNVSGLKFVISKVKA 166
JCM1132T-slpA       NFGSENAGKTVTLGSANSNVKFTGTNSDNQTETNVSTLKVKLDQNGVASLTNVSIANVYA 167
JCM1120T-SLP        NFGSENAGKEITIGSANPNVTFTEKTGDQ----PASTVKVTLDQDGVAKLSSVQIKNVYA 155
CNRZ892-slpH1       NFGSENAGKEITIGSANPNVTFTEKTGDQ----PASTVKVTLDQDGVAKLSSVQIKNVYA 155
                    .**. **** :*:   :   ..             .  **.*** * ..  .. * :* *

LH2171-SLP          YDSANTNAVSFYDAKSGLVATQGSYMT-LAENGNLNVDALLKALNDKYEAMQFKDGSFQT 225
CP790-prtY          YDSANTNAVSFYDAKSGLVATQGSYMT-LAENGNLNVDALLKALNDKYEAMQFKDRSFQT 225
JCM1132T-slpA       INTTDNSNVNFYDVTSGATVTNGAVSVNADNQGQVNVANVVAAINSKYFAAQYADKKLNT 227
JCM1120T-SLP        IDTTYNSNVNFYDVTTGATVTTGAVSIDADNQGQLNITSVVAAINSKYFAAQYDKKQLTN 215
CNRZ892-slpH1       IDTTYNSNVNFYDVTTGATVTTGAVSIDADNQGQLNITSVVAAINSKYFAAQYDKKQLTN 215
                     ::: .. *.***..:* ..* *:      ::*::*:  :: *:*.** * *: . .: .

LH2171-SLP          VKVNTTADDVKAELEKAGIKVDAANNFEAPDTFTVTLNAKSDVNGKTASLPVVVTVPNGK 285
CP790-prtY          VKVNTTADDVKAELEKAGIKVDAANNFEAPDTFTVTLNAKSDVNGKTASLPVVVTVPNGK 285
JCM1132T-slpA       RTAN-TEDAIKAALKDQKIDVNSVGYFKAPHTFTVNVKATSNTNGKSATLPVVVTVPNVA 286
JCM1120T-SLP        VTFD-TETAVKDALKAQKIEVSSVGYFKAPHTFTVNVKATSNKNGKSATLPVTVTVPNVA 274
CNRZ892-slpH1       VTFD-TETAVKDALKAQKIEVSSVGYFKAPHTFTVNVKATSNKNGKSATLPVTVTVPNVA 274
                     . : *   :*  *:   *.*.:.. *:**.****.::*.*: ***:*:***.*****

LH2171-SLP          STVVPSQSKTIMHNAYYYDKDAKRVGTDKVTRYNAVTVAMNTTKLANGISYYEVIENGKA 345
CP790-prtY          STVVPSQSKTIMHNAYYYDKEAKRVGTDKVTRYNAVTVAMNTTKLANGISYYS-IENGKA 344
JCM1132T-slpA       EPTVASVSKRIMHNAYYYDKDAKRVGTDSVKRYNSVSVLPNTT-TINGKTYYQVVENGKA 345
JCM1120T-SLP        DPVVPSQSKTIMHNAYFYDKDAKRVGTDKVTRYNTVTVAMNTTKLANGISYYEVIENGKA 334
CNRZ892-slpH1       DPVVPSQSKTIMHNAYFYDKDAKRVGTDKVTRYNTVTVAMNTTKLANGISYYEVIENGKA 334
                    . .* * ** ******:***:*******.*.***:*:*  ***   ** :**. :*****

LH2171-SLP          TGKYINADNIDGTKRTLKHNAYVYESSKKRANKVVLKKGTEVTTYGNPYTFKNGKKYYKI 405
CP790-prtY          TGKYINADNIDGTKRTLKHNAYVYESSKKRANKVVLKKGTEVTTYGNPYTFKNGKKYYKI 404
JCM1132T-slpA       VDKYINAANIDGTKRTLKHNAYVYASSKKRANKVVLKKGEVVTTYGASYTFKNGQKYYKI 405
JCM1120T-SLP        TGKYINADNIDGTKRTLKHNAYVYKTSKKRANKVVLKKGTEVTTYGGSYKFKNGQRYYKI 394
CNRZ892-slpH1       TGKYINADNIDGTKRTLKHNAYVYKTSKKRANKVVLKKGTEVTTYGGSYKFKNGQRYYKI 394
                    ..***** **************** :*************  *****  *.****::****

LH2171-SLP          GADTKKTYVRVENFD      420
CP790-prtY          GADTKKTYVRVENFD      419
JCM1132T-slpA       GDNTDKTYVKVANFR      420
JCM1120T-SLP        GANTEKTYVKVANFE      409
CNRZ892-slpH1       GANTEKTYVKVANFE      409
                    * :*.****:* **
```

Supplementary Figure 3. Multiple alignment of deduced amino acid sequences. Amino acid sequences of the predicted mature forms of LH2171 SLP, *L. helveticus* CP790 prtY (accession no. BAA86287.1), *L. acidophilus* JCM1132^{T} slpA (accession no. CAA50535.1), *L. helveticus* JCM1120^{T} SLP (accession no. CAB46985.1), and *L. helveticus* CNRZ892 slpH1 (accession no. CAA62606.1) were analyzed with Clustal Omega.
